# Light at night exposure and risk of depression: a meta-analysis of observational studies

**DOI:** 10.7189/jogh.15.04304

**Published:** 2025-10-31

**Authors:** Xiaomeng Li, Sijia Li, Qiaoling Geng, Binhao Wang, Xian Guo, Siyao Yan, Juan Zhang, Jianning Cai, Jianghong Chen, Xiaolin Zhang

**Affiliations:** 1 Department of Epidemiology and Statistics, School of Public Health, Hebei Medical University, Hebei Province Key Laboratory of Environment and Human Health, Shijiazhuang, China; 2 Department of epidemic treating and preventing, Center for Disease Prevention and Control of Shijiazhuang city, Shijiazhuang, China; 3 Department of Ultrasound, The First Hospital of Hebei Medical University, Shijiazhuang, China

## Abstract

**Background:**

Depression is a common mental disorder, and emerging evidence suggests a link between light at night (LAN) exposure and increased risk. This meta-analysis systematically synthesises the accumulating evidence on the LAN-depression relationship.

**Methods:**

We systematically examined PubMed, Web of Science, Embase, Cochrane Library, CNKI and Weipu databases up to June 2025, following PRISMA guidelines. The combined effect size was calculated based on a random-effects model. Heterogeneity among studies was assessed through Cochran's Q test and *I*^2^ statistic. Subgroup analyses were performed on types of LAN exposure, participant age categories, sample size, LAN exposure assessment and geographical regions. The study was registered on the international prospective register of systematic reviews (PROSPERO ID# CRD420251120552).

**Results:**

We included eight studies published from 2013–2025. Individuals with higher exposure to LAN had higher odds of depression (odds ratio (OR) = 1.224; 95% confidence interval (CI) = 1.176–1.275). Subgroup analyses revealed that the association varied by LAN exposure types, participant age categories, sample size, LAN exposure assessment and geographical regions.

**Conclusion:**

s Our research confirms a significant association between LAN exposure and elevated depression risk. Moderate to high heterogeneity and low sample sizes warrant higher-quality studies to help guide decisions to mitigate nocturnal light pollution and its psychological impacts.

Globally, we are experiencing unprecedented environmental changes concurrent with technological advancements. Electric lighting became widespread in the 20th century, introducing societies for the first time to bright artificial light at night (LAN) [1]. The presence of LAN enables socially valuable activities (*e.g*. social interaction, work, and leisure pursuits) to continue uninterrupted after dark [2]. As the world's industry grows and the population increases, the prevalence of both indoor and outdoor LAN continues to escalate. Outdoor sources encompass streetlamps and commercial displays, while indoor sources range from ambient lighting to emissions from digital devices (*e.g*. computers, smartphones).

Notably, Earth's total nighttime brightness is increasing at a rate of 1.8% per year [3], with China exhibiting the fastest growth rate (6.48% per year) and the USA maintaining the highest absolute brightness levels [4]. Mounting evidence suggests that exposure to high-intensity LAN poses significant risk to human health. Specifically, LAN exposure has been associated with cardiometabolic diseases [5]. Additionally, there is an increased risk of cancer (especially breast cancer) [6], obesity [7], there is also evidence that LAN can negatively affect mental health and sleep quality [8]. Despite these consequences, light pollution remains an under-prioritised and under-recognised environmental issue.

Depression is a debilitating mental disorder characterised by persistent low mood and loss of interest, recognised as the leading global cause of disability [9]. Clinical manifestations include anhedonia, appetite disturbances, sleep-wake cycle dysregulation, and in severe cases, self-harm or suicidal behaviours [10].

Currently, research on the link between LAN and mental health is rapidly accumulating, especially depression. One study suggests that exposure to brighter LAN is significantly associated with poorer mental health [11]. Another study reported no significant association between visible light at night and the incidence of depression [12]. These discrepancies may be attributed to differences in epidemiological design, population characteristics, and indoor/outdoor settings [13].

Given the inconsistencies in existing evidence and the growing prevalence of LAN exposure, we conducted the first meta-analysis to clarify the relationship between LAN and depression. Related results would be beneficial for the development of public health strategies aimed at mitigating the adverse effects of LAN on mental health.

## METHODS

### Literature search strategy

This meta-analysis was registered on PROSPERO (ID# CRD420251120552) and was reported in accordance with the PRISMA guidelines [14] (Table S1 in the **Online Supplementary Document**). A comprehensive literature search was performed across PubMed, Web of Science, Embase, the Cochrane Library, CNKI and Weipu databases for studies published up to June 2025. The search strategy incorporated Medical Subject Headings and free-text keywords related to LAN exposure and depression. To find articles analysing the connection between LAN exposure and depression, we employed a two-stage search approach. First, keywords associated with the relevant exposure and outcome variables were identified. Second, we manually screened the references lists of relevant articles to identify additional eligible studies. All of the retrieved records were imported into EndNote X9.

### Eligibility standards and research choice

#### Inclusion criteria

Studies were included based on the following predefined eligibility criteria:

1. Studies conducted in the general population (irrespective of age, sex or region) were included [5].

2. The exposure of interest included LAN and other light-emitting devices, such as self-reported interior LAN, photometric devices evaluated in studies, and satellite-measured outdoor LAN, were considered as the exposure of interest.

3.The comparator group consisted of participants with minimal exposure to LAN, low reported exposure levels, or no exposure to LAN (both indoors and outdoors).

4. Studies that characterised depression using standardised diagnostic to determine depressive outcomes were included.

5. Observational research designs, including case-control, cohort, and cross-sectional studies, were included.

#### Exclusion criteria

Studies were excluded according to the following criteria:

1. Non-human studies (*e.g*. animal or cell studies) were excluded.

2. Studies focusing on non-relevant exposures (*e.g*. early light exposure) were excluded.

3. Studies without relevant estimates or insufficient data to calculate such estimates were excluded.

4. Studies lacking standardised diagnostic criteria for depression outcomes were excluded.

5. Reviews, letters, conference abstracts, laboratory-based research, and commentaries were excluded.

### Data extraction

From each eligible study, two investigators (LXM and LSJ) retrieved the following information for each included study: first author's name, publication year, geographic area, sample size, study design, LAN exposure assessment method, outcome assessment method, adjusted variables, and study findings. Any discordance in data extraction was resolved through discussion and consensus.

### Literature quality evaluation

For cross-sectional studies, we evaluated seven studies using the Agency for Healthcare Research and Quality assessment criteria [15], which consist of 11 items, with a maximum possible score of 11. Each item answered ‘Yes’ was scored as 1, with higher scores indicating better study quality. Scores between 0–3 represent poor quality, 4–7 moderate quality, and scores between 8–11 excellent quality. For cohort studies, the quality was assessed with the Newcastle Ottawa Scale [16]. This system evaluates three domains: Selection, Comparability, and Exposure; on a scale of 0–9 scores, with higher scores indicating superior quality. Scores of 0–3 were considered poor quality, 4–6 moderate quality, and 7–9 excellent quality. All studies were evaluated by two authors separately. Disagreements were resolved through discussion, and if consensus could not be reached, a third author provided adjudication. Each study was ultimately classified as high, moderate, or low quality.

### Statistical analysis method

Given the anticipated heterogeneity across studies (*e.g*. sample size, age and other confounding variables), we utilised a random-effects model for meta-analysis to compare the pooled effects of the highest *vs*. lowest levels of LAN exposure. The extracted odds ratio (OR) was derived from the most fully adjusted model reported in each study.

Cochran's Q test and *I*^2^ statistic were used to assess the heterogeneity across studies. In this analysis, *I*^2^>50% combined with *P* < 0.1 were considered indicative of significant heterogeneity [17].

Subgroup analyses were conducted based on key characteristics of the included studies. These analyses focused on two primary dimensions:

1. extrinsic factors (*e.g*. geographical location: Asia *vs*. non-Asia)

2. topic-related factors (*e.g*. LAN type: indoor *vs*. outdoor; LAN exposure assessment: light meter *vs*. satellite; age stratification <65 years *vs*. ≥65 years; sample size ≤2000 *vs*. >2000).

Sensitivity analyses were performed by sequentially omitting individual studies to evaluate the robustness of the pooled estimates and to identify studies exerting disproportionate influence on heterogeneity. Publication bias was assessed through visual inspection of funnel plots and further tested using Egger’s regression method.

All statistical analyses were conducted using Stata 15.0 (StataCorp LLC, College Station, TX, USA).

## RESULTS

### Literature search

**Figure 1:**
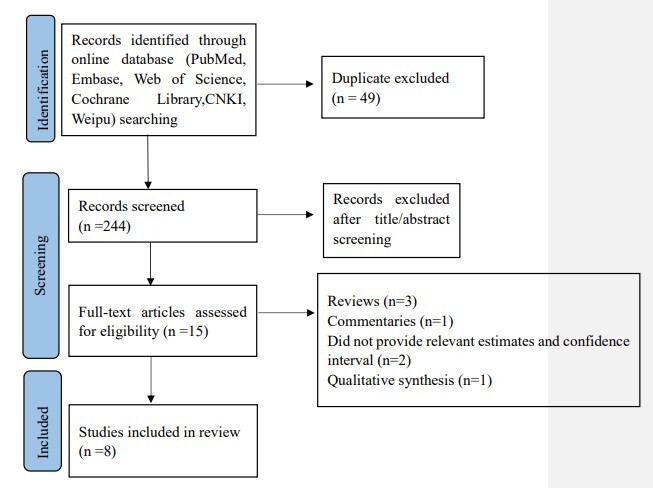
Flowchart of literature selection process.

In total, 293 articles were retrieved from the following databases: PubMed (106 articles), Web of Science (23 articles), Embase (92 articles), Cochrane Library (six articles), CNKI (67 articles) and Weipu (three articles). After removing duplicates (49 articles), we screened 244 titles and abstracts. Subsequently, 15 full-text articles were assessed for eligibility. Of these, eight studies were excluded (**Figure 1**) due to the following reasons:

1) reviews or commentaries

2) lacking relevant estimates with confidence intervals (CIs)

3) based solely on qualitative synthesis.

Ultimately, eight studies were deemed suitable for inclusion.

### Study characteristics

This study included eight research papers published from 2013–2025, with a total sample size of 205 906 individuals. The geographic distribution of the studies was as follows: one study from North America [18], two from Europe [19,20], five from Asia [21–25]. Regarding LAN exposure evaluation, four studies (50.0%) gathered data on LAN exposure outdoors, while four studies (50.0%) assessed LAN exposure indoor (**Table 1**).

**Table 1:** Summary of key features

Author (year), region	Sample size (age range), design	LAN exposure assessment	Outcomes assessment	Whether influencing factors	Main results (OR)
MIN (2018), Korean [21]	113 119 (≥20), cross-sectional	Outdoor Satellite	CES-D	Yes	1.29 (95% CI = 1.15–1.46)
ZHU (2024), China [22]	6445 (≥60), cross-sectional	Outdoor Satellite	CES-D	Yes	1.49 (95% CI = 1.15–1.92)
OBAYASHI (2013), Japan [24]	516 (≥60) cross-sectional	Indoor Light meter	GDS-15	Yes	1.89 (95% CI = 1.10–3.25)
PAKSARIAN (2020), USA [18]	10 123 (13–18) cross-sectional	Outdoor Satellite	DSM-IV	Yes	1.07 (95% CI = 1.00–1.15)
BURNS (2022), UK [20]	86 772 (40–69) cross-sectional	Indoor light meter	PHQ-9	Yes	1.30 (95% CI = 1.23–1.38)
OBAYASHI (2022), Japan [23]	2947 (>40) cross-sectional	Indoor light meter	GDS-15	Yes	1.33 (95% CI = 1.00–1.77)
OBAYASHI (2018), Japan [25]	1127 (≥60) Longitudinal study	Indoor light meter	GDS-15	Yes	1.72 (95% CI = 1.03–2.89)
HELBICH (2022), Netherlands [19]	10 482 (18–65), cross-sectional	Outdoor Satellite	PHQ-9	Yes	1.72 (95% CI = 1.15–2.56)

CES-D – Center for Epidemiologic Studies Depression Scale, DSM-IV – fourth edition of the Diagnostic and Statistical Manual of Mental Disorders, GDS-15 – Geriatric Depression Scale, PHQ-9 – Patient Health Questionnaire

All studies clearly described the methods for assessing LAN exposure and depression outcomes. Adjusted estimates were available for analysis after accounting for potential confounding factors.

### Quality assessment

Among the eight included studies, seven were cross-sectional studies and all scored ≥5 points on the Agency for Healthcare Research and Quality scale, indicating moderate to high methodological quality. The remaining study received a score of seven on the Newcastle-Ottawa Scale, reflecting high-quality research.

### Exposure to LAN during the night and depression

**Figure 2:**
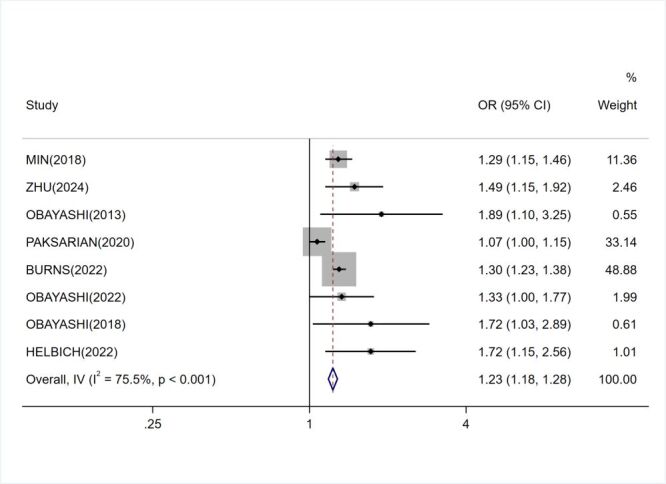
Meta-analysis for link of LAN exposure and depression risk. CI – confidence interval, LAN – light at night, OR – odds ratio.

Pooled analysis of eight studies revealed a weak link between LAN exposure and elevated risk of depression (OR = 1.224; 95% CI = 1.176–1.275), with substantial heterogeneity (*I*^2^ = 75.7%, *P* < 0.001) (**Figure 2**). The substantial heterogeneity indicates significant variability across studies.

**Figure 3:**
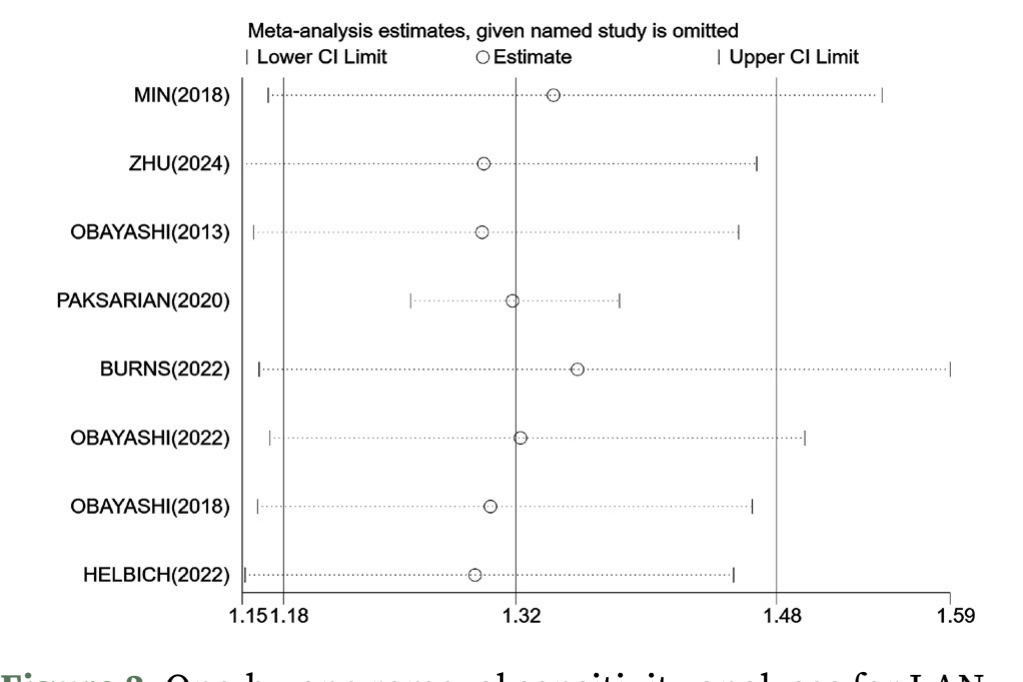
One-by-one removal sensitivity analyses for LAN exposure and depression risk. CI – confidence interval, LAN – light at night.

Sensitivity analyses demonstrated that the robustness of pooled estimates was not affected by the exclusion of any single study, as the overall conclusions remained unchanged (**Figure 3**).

Visual inspection of the funnel plot did not reveal any significant publication bias (Figure S1 in the **Online Supplementary Document**), which was consistent with the results of our Egger’s regression test (*P* = 0.174).

### Subgroup analysis

Subgroup analyses suggested potential sources of heterogeneity (**Table 2**). For LAN type/exposure assessment, outdoor LAN (satellite-based) was significantly associated with depression risk (OR = 1.149; 95% CI = 1.084–1.218, *P* = 0.001), whereas indoor LAN (light meter–based) was not (OR = 1.311; 95% CI = 1.239–1.386, *P* = 0.406). By region, studies conducted outside Asia showed a significant association (OR = 1.207; 95% CI = 1.155–1.261, *P* < 0.001), while those from Asia did not (OR = 1.352; 95% CI = 1.227–1.491, *P* = 0.483). Stratified by age, studies including participants <65 years reported significant associations (OR = 1.217; 95% CI = 1.167–1.268, *P* < 0.001), whereas those with participants ≥65 years did not (OR = 1.488; 95% CI = 1.256–1.764, *P* = 0.649). Similarly, studies with larger sample sizes (>2000) showed significant associations (OR = 1.225; 95% CI = 1.176–1.276, *P* < 0.001), while smaller studies (≤2000) did not (OR = 1.799; 95% CI = 1.238–2.614, *P* = 0.805).

**Table 2:** Subgroup analyses by LAN type, region and age

Group		No. of estimates					Effect estimates OR (95% CI)			*P-*value		*I* ^2^	
LAN type/exposure assessment													
*Outdoor/satellite*		4					1.149 (1.084–1.218)			0.001		80.6%	
*Indoor/light meter*		4					1.311 (1.239–1.386)			0.406		0.0%	
Region													
*Asia*		5					1.352 (1.227–1.491)			0.483		0.0%	
*Not Asia*		3					1.207 (1.155–1.261)			<0.001		90.4%	
Age													
*≥65 y*		4					1.488 (1.256–1.764)			0.649		0.0%	
*<65 y*		4					1.217 (1.167–1.268)			<0.001		86.3%	
Sample size													
*≤2000*		2					1.799 (1.238–2.614)			0.805		0.0%	
*>2000*		6					1.225 (1.176–1.276)			<0.001		79.6%	

CI – confidence interval, LAN – light at night, OR – odds ratio

## DISCUSSION

Our meta-analysis, comprising eight observational studies with a total sample size of 205 906 participants, found that higher exposure to LAN was associated with an elevated risk of depression (OR = 1.224; *P* < 0.001). Despite the effect size indicates only a weak association, its potential public health impact is considerable. Given the ubiquity of LAN in modern societies, even small increases in individual risk could translate into a considerable additional burden of depression at the population level. These findings highlight the need to consider nocturnal light pollution as a modifiable environmental risk factor with important implications for mental health prevention strategies.

Subgroup analyses showed higher heterogeneity among studies examining outdoor LAN assessed via satellite compared with those focusing on indoor LAN via light meter, this suggests that the method of LAN exposure assessment likely contributed to the heterogeneity in the overall analysis. Satellite-derived measures often estimate light levels across residential areas or buffer zones, which may not fully capture individual-level exposure. In contrast, indoor LAN exposure can be measured more directly, but such studies are often limited by smaller sample sizes given the complexity of objective indoor assessments.

Geographic differences were also evident. Studies conducted in Asian regions yielded non-significant but more consistent results, whereas those from non-Asian regions demonstrated significant associations accompanied by high heterogeneity. Since the current meta-analysis included research from only six countries across three regions (Asia, North America, and Europe), the limited geographic coverage highlights the need for studies in more racially, ethnically, and geographically diverse populations to strengthen the generalisability.

In subgroup analyses, age may also have contributed to heterogeneity. Studies including younger participants showed significant associations but also higher heterogeneity, whereas those focusing on older adults showed no significant association and minimal heterogeneity. Future research should include populations across different age groups to further investigate the relationship between nighttime light exposure and depression. Collectively, these findings underscore the importance of conducting large-scale, well-designed cohort studies that objectively and rigorously assess LAN exposure to clarify its relationship with depression.

Accelerated urbanisation poses risks to mental health and well-being [26], while LAN affects not only humans but also entire ecosystems [27]. Light is the strongest zeitgeber (from German, ‘time giver’) for the human circadian system [28], and LAN exposure may disrupt circadian rhythms by inhibiting melatonin secretion through retinal circuits, thereby the elevating risk of sleep problems [29,30]. Existing evidence further supports links between sleep disturbances and depression [31],and circadian dysregulation is consistently observed in adults with depressive disorders and correlates with core clinical symptoms [32]. A systematic review [33]concluded that prolonged LAN exposure primarily triggers circadian disruption, mood alterations, and depressive phenotypes. Animal studies provide mechanistic insights that complement human findings. Evidence from animal models suggests that LAN exposure can lead to depressive-like behaviours, potentially mediated through glucocorticoid-dependent pathway and hippocampal gene expression changes [34]. Despite these findings, the precise mechanisms linking LAN to depression risk require further elucidation.

To our knowledge, this study is the first meta-analysis to quantitatively explore the association between LAN and depression risk. Our findings provide preliminary evidence that LAN may represent a modifiable risk factor for depression, offering a foundation for future mechanistic and longitudinal investigations.

In order to properly interpret the results, several limitations of this meta-analysis must be acknowledged. First, the number of eligible studies was small (fewer than 10), which restricts statistical power. Second, similar to prior research on LAN and disease associations, substantial heterogeneity was observed, likely due to variability in study designs, LAN types, measurement methods, exposure timing, and inconsistent criteria for defining depression. Residual or unmeasured confounders may also have influenced the estimates. Third, the current analysis could not evaluate potential nonlinear relationships between LAN exposure and depression risk. Fourth, the included studies involved 205 906 participants, which constitutes a relatively small sample size and limits generalisability. Seven were cross-sectional, while only one employed a longitudinal design. Robust causal inferences thus require future studies with more rigorous methodologies, particularly longitudinal cohorts. Finally, generalisability is limited as all studies were conducted in upper-middle-income countries across Asia, Europe, and North America, leaving the effects in other populations uncertain. The exclusion of non-English publications may have resulted in the omission of relevant data.

The current meta-analysis identified a positive association between elevated LAN exposure and an increased risk of depression. These findings may guide researchers in designing future studies and assist policymakers in developing urban infrastructure strategies to mitigate depression risks. However, limitations such as the scarcity of included studies, substantial heterogeneity, and potential bias preclude definitive conclusions regarding the LAN-depression relationship. Future research incorporating higher-quality evidence and advanced quantitative analyses is warranted to elucidate this association further.

## CONCLUSIONS

Our meta-analysis provides preliminary evidence that higher exposure to LAN is associated with an increased risk of depression. Although the observed association is modest, its potential public health implications are substantial given the widespread presence of LAN in modern societies. Future large-scale, longitudinal, and methodologically rigorous studies across diverse populations are needed to clarify the causal pathways and inform public health strategies aimed at mitigating the mental health risks of nocturnal light pollution.

## Additional material


Online Supplementary Document
