# Supplementary material for: Light at night exposure and risk of depression: a meta-analysis of observational studies
Source: J Glob Health. 2025 Oct 31;15:04304. doi: 10.7189/jogh.15.04304 (PMC12576862; doi:10.7189/jogh.15.04304)
Supplement: Online Supplementary Document [file jogh-15-04304-s001.pdf]

Supplement to: Li X, Li S, Geng Q, Wang B, Guo X, Yan S, Zhang J, Cai J, Chen J, Zhang X. Light at night exposure and risk of depression: a meta-analysis of observational studies. J Glob Health. 2025;15:04304.

Light at Night Exposure and Risk of Depression: A Me-ta-Analysis of Observational Studies Contents

File S1 The detailed search strategy

Table S1. PRISMA Checklist

Table S2. Critical appraisal of the included studies based on the Agency for Healthcare Research and Quality (AHRQ) scale

Figure S1. The bias testing results for LAN exposure and depression. LAN – light at night.

Web of Science 23 results

Searches:

1: (((ALL=(Light pollution)) OR ALL=(Pollution, Light or Artificial Light at Night or Artificial Night Lighting or Artificial Night Lightings or Lighting, Artificial Night or Lightings, Artificial Night or Night Lighting, Artificial or Night Lightings, Artificial or Skyglow or Artificial Night Sky Brightness)) AND ALL=(depression)) OR ALL=(Depressive Symptoms or Symptom, Depressive or Depressive Symptom or Depression, Emotional or Emotional Depression) Results: 111598

2: (ALL=(Light pollution or Pollution, Light or Artificial Light at Night or Artificial Night Lighting or Artificial Night Lightings or Lighting, Artificial Night or Lightings, Artificial Night or Night Lighting, Artificial or Night Lightings, Artificial or Skyglow or Artificial Night Sky Brightness)) AND ALL=(Dpressive or Depressive Symptoms or Symptom, Depressive or Depressive Symptom or Depression, Emotional or Emotional Depression) Results: 23

3: (ALL=(Light pollution or Pollution, Light or Artificial Light at Night or Artificial Night Lighting or Artificial Night Lightings or Lighting, Artificial Night or Lightings, Artificial Night or Night Lighting, Artificial or Night Lightings, Artificial or Skyglow or Artificial Night Sky Brightness)) AND ALL=(Dpressive or Depressive Symptoms or Symptom, Depressive or Depressive Symptom or Depression, Emotional or Emotional Depression) Results: 23

Cochrane library 6 results

ID Search Hits

#1 MeSH descriptor: [Light Pollution] explode all trees 1

#2 (Skyglow or Artificial Night Sky Brightness or Pollution, Light or Lightings, Artificial Night or Artificial Night Lightings or Lighting, Artificial Night or Night Lightings, Artificial or Artificial Light at Night or Night Lighting, Artificial or Artificial Night Lighting):ti,ab,kw (Word variations have been searched) 155

#3 #1 or #2 155

#4 MeSH descriptor: [Depression] explode all trees 17992

#5 (Depressive Symptoms or Symptom, Depressive or Depressive Symptom or Depression, Emotional or Emotional Depression):ti,ab,kw (Word variations have been searched) 49581

#6 #4 or #5 57575

#7 #3 and #66

PubMed 102results

1 Query "Light Pollution"[Majr] Sort By Most Recent Search details "Light Pollution"[MeSH Major Topic] Results 81

2 Query Pollution, Light or Artificial Light at Night or Artificial Night Lighting or Artificial Night Lightings or Lighting, Artificial Night or Lightings, Artificial Night or Night Lighting, Artificial or Night Lightings, Artificial or Skyglow or Artificial Night Sky Brightness Search details "light pollution"[MeSH Terms] OR ("light"[All Fields] AND "pollution"[All Fields]) OR "light pollution"[All Fields] OR ("pollution"[All Fields] AND "light"[All Fields]) OR "pollution light"[All Fields] OR ("light pollution"[MeSH Terms] OR ("light"[All Fields] AND "pollution"[All Fields]) OR "light pollution"[All Fields] OR ("artificial"[All Fields] AND "light"[All Fields] AND "night"[All Fields]) OR "artificial light at night"[All Fields]) OR ("light pollution"[MeSH Terms] OR ("light"[All Fields] AND "pollution"[All Fields]) OR "light pollution"[All Fields] OR ("artificial"[All Fields] AND "night"[All Fields] AND "lighting"[All Fields]) OR "artificial night lighting"[All Fields]) OR ("light pollution"[MeSH Terms] OR ("light"[All Fields] AND "pollution"[All Fields]) OR "light pollution"[All Fields] OR ("artificial"[All Fields] AND "night"[All Fields] AND "lightings"[All Fields])) OR ("light pollution"[MeSH Terms] OR ("light"[All Fields] AND "pollution"[All Fields]) OR "light pollution"[All Fields] OR ("lighting"[All Fields] AND "artificial"[All Fields] AND "night"[All Fields])) OR ("light pollution"[MeSH Terms] OR ("light"[All Fields] AND "pollution"[All Fields]) OR "light pollution"[All Fields] OR ("lightings"[All Fields] AND "artificial"[All Fields] AND "night"[All Fields])) OR ("light pollution"[MeSH Terms] OR ("light"[All Fields] AND "pollution"[All Fields]) OR "light pollution"[All Fields] OR ("night"[All Fields] AND "lighting"[All Fields] AND "artificial"[All Fields])) OR ("light pollution"[MeSH Terms] OR ("light"[All Fields] AND "pollution"[All Fields]) OR "light pollution"[All Fields] OR ("night"[All Fields] AND "lightings"[All Fields] AND "artificial"[All Fields])) OR ("light pollution"[MeSH Terms] OR ("light"[All Fields] AND "pollution"[All Fields]) OR "light pollution"[All Fields] OR "skyglow"[All Fields]) OR ("light pollution"[MeSH Terms] OR ("light"[All Fields] AND "pollution"[All Fields]) OR "light pollution"[All Fields] OR

("artificial"[All Fields] AND "night"[All Fields] AND "sky"[All Fields] AND "brightness"[All Fields]) OR "artificial night sky brightness"[All Fields]) Results 10,341

3 Query "Depressive Disorder"[Mesh] Sort By Most Recent Search details "Depressive Disorder"[MeSH Terms] Results 123,995

4 Query Depressive Symptoms or Depressive Symptom or Symptom, Depressive or Depression or Depression, Emotional Search details "depression"[MeSH Terms] OR "depression"[All Fields] OR ("depressive"[All Fields] AND "symptoms"[All Fields]) OR "depressive symptoms"[All Fields] OR ("depression"[MeSH Terms] OR "depression"[All Fields] OR ("depressive"[All Fields] AND "symptom"[All Fields]) OR "depressive symptom"[All Fields]) OR ("depression"[MeSH Terms] OR "depression"[All Fields] OR ("symptom"[All Fields] AND "depressive"[All Fields]) OR "symptom depressive"[All Fields]) OR ("depressed"[All Fields] OR "depression"[MeSH Terms] OR "depression"[All Fields] OR "depressions"[All Fields] OR "depression s"[All Fields] OR "depressive disorder"[MeSH Terms] OR ("depressive"[All Fields] AND "disorder"[All Fields]) OR "depressive disorder"[All Fields] OR "depressivity"[All Fields] OR "depressive"[All Fields] OR "depressively"[All Fields] OR "depressiveness"[All Fields] OR "depressives"[All Fields]) OR ("depression"[MeSH Terms] OR "depression"[All Fields] OR ("depression"[All Fields] AND "emotional"[All Fields]) OR "depression emotional"[All Fields]) Results 637,747

5 Query ("Light Pollution"[Majr]) OR (Pollution, Light or Artificial Light at Night or Artificial Night Lighting or Artificial Night Lightings or Lighting, Artificial Night or Lightings, Artificial Night or Night Lighting, Artificial or Night Lightings, Artificial or Skyglow or Artificial Night Sky Brightness) Search details "Light Pollution"[MeSH Major Topic] OR ("Light Pollution"[MeSH Terms] OR ("light"[All Fields] AND "pollution"[All Fields]) OR "Light Pollution"[All Fields] OR ("pollution"[All Fields] AND "light"[All Fields]) OR "pollution light"[All Fields] OR ("Light Pollution"[MeSH Terms] OR ("light"[All Fields] AND "pollution"[All Fields]) OR "Light Pollution"[All Fields] OR ("artificial"[All Fields] AND "light"[All Fields] AND "night"[All Fields]) OR "artificial light at night"[All Fields]) OR ("Light Pollution"[MeSH Terms] OR ("light"[All Fields] AND "pollution"[All Fields]) OR "Light Pollution"[All Fields] OR ("artificial"[All Fields] AND "night"[All Fields] AND "lighting"[All Fields]) OR "artificial night lighting"[All Fields]) OR ("Light Pollution"[MeSH Terms] OR ("light"[All Fields] AND "pollution"[All Fields]) OR "Light Pollution"[All Fields] OR ("artificial"[All Fields] AND "night"[All Fields] AND "lightings"[All Fields])) OR ("Light Pollution"[MeSH Terms] OR ("light"[All Fields] AND "pollution"[All Fields]) OR "Light Pollution"[All Fields] OR ("lighting"[All Fields] AND "artificial"[All Fields] AND "night"[All Fields])) OR ("Light Pollution"[MeSH Terms] OR ("light"[All Fields] AND "pollution"[All Fields]) OR "Light Pollution"[All Fields] OR ("lightings"[All Fields] AND "artificial"[All Fields] AND "night"[All Fields])) OR ("Light Pollution"[MeSH Terms] OR ("light"[All Fields] AND "pollution"[All Fields]) OR "Light Pollution"[All Fields] OR ("night"[All Fields] AND "lighting"[All Fields] AND "artificial"[All Fields])) OR ("Light Pollution"[MeSH Terms] OR ("light"[All Fields] AND "pollution"[All Fields]) OR "Light Pollution"[All Fields] OR ("night"[All Fields] AND "lightings"[All Fields] AND "artificial"[All Fields])) OR ("Light Pollution"[MeSH Terms] OR ("light"[All Fields] AND "pollution"[All Fields]) OR "Light Pollution"[All Fields] OR "skyglow"[All Fields]) OR ("Light Pollution"[MeSH Terms] OR ("light"[All Fields] AND "pollution"[All Fields]) OR "Light Pollution"[All Fields] OR ("artificial"[All Fields] AND "night"[All Fields] AND "sky"[All Fields] AND "brightness"[All Fields]) OR "artificial night sky brightness"[All Fields])) Results 10,341

6 Query ("Depressive Disorder"[Mesh]) OR (Depressive Symptoms or Depressive Symptom or Symptom, Depressive or Depression or Depression, Emotional)Search details "Depressive Disorder"[MeSH Terms] OR ("depression"[MeSH Terms] OR "depression"[All Fields] OR ("depressive"[All Fields] AND "symptoms"[All Fields]) OR "depressive symptoms"[All Fields] OR ("depression"[MeSH Terms] OR "depression"[All Fields] OR ("depressive"[All Fields] AND "symptom"[All Fields]) OR "depressive symptom"[All Fields]) OR ("depression"[MeSH Terms] OR "depression"[All Fields] OR ("symptom"[All Fields] AND "depressive"[All Fields]) OR "symptom depressive"[All Fields]) OR ("depressed"[All Fields] OR "depression"[MeSH Terms] OR "depression"[All Fields] OR "depressions"[All Fields] OR "depression s"[All Fields] OR "Depressive Disorder"[MeSH Terms] OR ("depressive"[All Fields] AND "disorder"[All Fields]) OR "Depressive Disorder"[All Fields] OR "depressivity"[All Fields] OR "depressive"[All Fields] OR "depressively"[All Fields] OR "depressiveness"[All Fields] OR "depressives"[All Fields]) OR ("depression"[MeSH Terms] OR "depression"[All Fields] OR ("depression"[All Fields] AND "emotional"[All Fields]) OR "depression emotional"[All Fields])) Results 637,74

7 Query (("Light Pollution"[Majr]) OR (Pollution, Light or Artificial Light at Night or Artificial Night Lighting or Artificial Night Lightings or Lighting, Artificial Night or Lightings, Artificial Night or Night Lighting, Artificial or Night Lightings, Artificial or Skyglow or Artificial Night Sky Brightness)) AND (("Depressive Disorder"[Mesh]) OR (Depressive Symptoms or Depressive Symptom or Symptom, Depressive or Depression or Depression, Emotional)Search details ("Light Pollution"[MeSH Major Topic] OR ("Light Pollution"[MeSH Terms] OR ("light"[All Fields] AND "pollution"[All Fields]) OR "Light Pollution"[All Fields] OR ("pollution"[All Fields] AND "light"[All Fields]) OR "pollution light"[All Fields] OR ("Light Pollution"[MeSH Terms] OR ("light"[All Fields] AND "pollution"[All Fields]) OR "Light Pollution"[All Fields] OR ("artificial"[All Fields] AND "light"[All Fields] AND "night"[All Fields]) OR "artificial light at night"[All Fields]) OR ("Light Pollution"[MeSH Terms] OR ("light"[All Fields] AND "pollution"[All Fields]) OR "Light Pollution"[All Fields] OR ("artificial"[All Fields] AND "night"[All Fields] AND "lighting"[All Fields]) OR "artificial night lighting"[All Fields]) OR ("Light Pollution"[MeSH Terms] OR ("light"[All Fields] AND "pollution"[All Fields]) OR "Light Pollution"[All Fields] OR ("artificial"[All Fields] AND "night"[All Fields] AND "lightings"[All Fields])) OR ("Light Pollution"[MeSH Terms] OR ("light"[All Fields] AND "pollution"[All Fields]) OR "Light Pollution"[All Fields] OR ("lighting"[All Fields] AND "artificial"[All Fields] AND "night"[All Fields])) OR ("Light Pollution"[MeSH Terms] OR ("light"[All Fields] AND "pollution"[All Fields]) OR "Light Pollution"[All Fields] OR ("lightings"[All Fields] AND "artificial"[All Fields] AND "night"[All Fields])) OR ("Light Pollution"[MeSH Terms] OR ("light"[All Fields] AND "pollution"[All Fields]) OR "Light Pollution"[All Fields] OR ("night"[All Fields] AND "lighting"[All Fields] AND "artificial"[All Fields])) OR ("Light Pollution"[MeSH Terms] OR ("light"[All Fields] AND "pollution"[All Fields]) OR "Light Pollution"[All Fields] OR ("night"[All Fields] AND "lightings"[All Fields] AND "artificial"[All Fields])) OR ("Light Pollution"[MeSH Terms] OR ("light"[All Fields] AND "pollution"[All Fields]) OR "Light Pollution"[All Fields] OR "skyglow"[All Fields]) OR ("Light Pollution"[MeSH Terms] OR ("light"[All Fields] AND "pollution"[All Fields]) OR "Light Pollution"[All Fields] OR ("artificial"[All Fields] AND "night"[All Fields] AND "sky"[All Fields] AND "brightness"[All Fields]) OR "artificial night sky brightness"[All Fields]))) AND ("Depressive Disorder"[MeSH Terms] OR ("depression"[MeSH Terms] OR "depression"[All Fields] OR ("depressive"[All Fields] AND "symptoms"[All Fields]) OR "depressive symptoms"[All Fields] OR ("depression"[MeSH Terms] OR "depression"[All Fields] OR ("depressive"[All Fields] AND "symptom"[All Fields]) OR "depressive symptom"[All Fields]) OR ("depression"[MeSH Terms] OR "depression"[All Fields] OR ("symptom"[All Fields] AND "depressive"[All Fields]) OR "symptom depressive"[All Fields]) OR ("depressed"[All Fields] OR "depression"[MeSH Terms] OR "depression"[All Fields] OR "depressions"[All Fields] OR "depression s"[All Fields] OR "Depressive Disorder"[MeSH Terms] OR ("depressive"[All Fields] AND "disorder"[All Fields]) OR "Depressive Disorder"[All Fields] OR "depressivity"[All Fields] OR "depressive"[All Fields] OR "depressively"[All Fields] OR "depressiveness"[All Fields] OR "depressives"[All Fields]) OR ("depression"[MeSH Terms] OR "depression"[All Fields] OR ("depression"[All Fields] AND "emotional"[All Fields]) OR "depression emotional"[All Fields]))) Results 106

Embase 92 results

Session Results

No.	Query Results	Results
#7.	#3 AND #6	92
#6.	#4 OR #5	896,659
#5.	depression:ab,ti OR 'clinical depression':ab,ti OR 'depressive disorder':ab,ti OR 'depressive illness':ab,ti OR 'depressive symptom':ab,ti OR 'mental depression':ab,ti	619,307
#4.	'depression'/exp	670,130
#3.	#1 OR 2	1,621
#2.	'light at night':ab,ti OR 'artificial light at night':ab,ti OR 'artificial night sky brightness':ab,ti OR 'artificial night lighting':ab,ti OR 'artificial night lightings':ab,ti	1,336
#1.	'light pollution'/exp	503

CNKI: 67 articles

Retrieval conditions: ((((Subject=Depressive Symptoms or title=Depressive Symptoms or v_subject=Chinese and English extension (Depressive Symptoms) or title=Chinese and English extension (Depressive Symptom)) or ((subject=Depressive Symptom or Title=Depressive Symptom or v_subject=Chinese and English Extended (Depressive Symptom) or title=Chinese and English Extended (Symptom, Depressive)) or (Subject=Symptom, Depressive or Title=Symptom, Depressive or v_subject=Chinese and English Extended (Symptom, Depressive) or title=Chinese and English Extended (Symptom, Depressive)))) or ((subject=Emotional Depression or title=Emotional Depression or v_subject=Chinese and English Extended (Emotional Depression) or title=Chinese and English Extended (Emotional Depression)) or (subject=Depression, Emotional or title=Depression, Emotional or v_subject=extended Chinese and English (Depression, Emotional) or title=extended Chinese and English (Depression, Emotional)))) and ((subject=Pollution, Light or title=Pollution, Light or v_subject=Chinese and English extension (Pollution, Light) or title=Chinese and English extension (Pollution, Light)) or (subject=Artificial Light at Night or title=Artificial Light at Night or v_subject=Chinese and English Extended (Artificial Light at Night) or title=Chinese and English extended (Artificial Light at Night)) or (subject=Artificial Night Lighting or title=Artificial Night Lighting or v_subject=Chinese and English Extended (Artificial Night Lighting) or title=Chinese and English extended (Artificial Night Lighting)) or (subject=Artificial Night Lightings or title=Artificial Night Lightings or v_subject=Chinese and English Extended (Artificial Night Lightings) or title=Chinese and English extended (Artificial Night Lightings)) or (subject=Lighting, Artificial Night or title=Lighting, Artificial Night or v_subject=Chinese and English Extended (Lighting, Artificial Night) or title=Chinese and English extended (Lighting, Artificial Night)) or (subject=Lightings, Artificial Night or title=Lightings, Artificial Night or v_subject=Chinese and English Extended (Lightings, Artificial Night) or title=Chinese and English extended (Lightings, Artificial Night)) or (subject=Night Lighting, Artificial or title=Night Lighting, Artificial or v_subject=Chinese and English Extended (Night Lighting, Artificial) or title=Chinese and English extended (Night Lighting, Artificial)) or (subject=Night Lightings, Artificial or title=Night Lightings, Artificial or v_subject=Chinese and English Extended (Night Lightings) or title=Chinese and English extended (Night Lightings)) or (subject=Skyglow or title=Skyglow or v_subject=Chinese and English Extended (Skyglow) or title=Chinese and English extended (Skyglow)) or (subject=Artificial Night Sky Brightness or title=Artificial Night Sky Brightness or v_subject=Chinese and English Extended (Artificial Night Sky Brightness) or title=Chinese and English extended (Artificial Night Sky Brightness)))) (fuzzy matching), album navigation: all; database: literature cross-database search

Search method: cross-database search

Database: Literature

Weipu-------3 article

Any field = Pollution, Light OR Artificial Light at Night OR Artificial Night Lighting OR Artificial Night Lightings OR Lighting, Artificial Night OR Lightings, Artificial Night OR Night Lighting, Artificia OR Night Lightings, Artificial OR Skyglow ORArtificial Night Sky Brightness AND Any field = Depressive Symptoms OR Depressive Symptom OR Symptom, Depressive OR Emotional Depression OR Depression, Emotional AND Year: 1990-2025

Section and Topic	Item #	Checklist item	Location where item is reported
**TITLE**			
Title	1	Identify the report as a systematic review.	1st page
**ABSTRACT**			
Abstract	2	See the PRISMA 2020 for Abstracts checklist.	2nd page
**INTRODUCTION**			
Rationale	3	Describe the rationale for the review in the context of existing knowledge.	3th page 1-23lines
Objectives	4	Provide an explicit statement of the objective(s) or question(s) the review addresses.	3th page 23-29lines and 4th page 1-6lines
**METHODS**			
Eligibility criteria	5	Specify the inclusion and exclusion criteria for the review and how studies were grouped for the syntheses.	4th page 20-29lines and 5th page 1-13lines
Information sources	6	Specify all databases, registers, websites, organisations, reference lists and other sources searched or consulted to identify studies. Specify the date when each source was last searched or consulted.	4thpage 8-13lines
Search strategy	7	Present the full search strategies for all databases, registers and websites, including any filters and limits used.	4th page 13-19lines
Selection process	8	Specify the methods used to decide whether a study met the inclusion criteria of the review, including how many reviewers screened each record and each report retrieved, whether they worked independently, and if applicable, details of automation tools used in the process.	5th page 19 line
Data collection process	9	Specify the methods used to collect data from reports, including how many reviewers collected data from each report, whether they worked independently, any processes for obtaining or confirming data from study investigators, and if applicable, details of automation tools used in the process.	5th page 15-16lines
Data items	10a	List and define all outcomes for which data were sought. Specify whether all results that were compatible with each outcome domain in each	6th page 8-

Section and Topic	Item #	Checklist item	Location where item is reported
		study were sought (e.g. for all measures, time points, analyses), and if not, the methods used to decide which results to collect.	10lines
	10b	List and define all other variables for which data were sought (e.g. participant and intervention characteristics, funding sources). Describe any assumptions made about any missing or unclear information.	6th page 8-10lines
Study risk of bias assessment	11	Specify the methods used to assess risk of bias in the included studies, including details of the tool(s) used, how many reviewers assessed each study and whether they worked independently, and if applicable, details of automation tools used in the process.	5th page 20-28lines and 6th page 1-4lines
Effect measures	12	Specify for each outcome the effect measure(s) (e.g. risk ratio, mean difference) used in the synthesis or presentation of results.	6th page 8-10lines
Synthesis methods	13a	Describe the processes used to decide which studies were eligible for each synthesis (e.g. tabulating the study intervention characteristics and comparing against the planned groups for each synthesis (item #5)).	Null
	13b	Describe any methods required to prepare the data for presentation or synthesis, such as handling of missing summary statistics, or data conversions.	Null
	13c	Describe any methods used to tabulate or visually display results of individual studies and syntheses.	6th page 6-8lines
	13d	Describe any methods used to synthesize results and provide a rationale for the choice(s). If meta-analysis was performed, describe the model(s), method(s) to identify the presence and extent of statistical heterogeneity, and software package(s) used.	6th page 11-13lines and 6th page23 line
	13e	Describe any methods used to explore possible causes of heterogeneity among study results (e.g. subgroup analysis, meta-regression).	6th page 14-18lines
	13f	Describe any sensitivity analyses conducted to assess robustness of the synthesized results.	6th page19-22lines
Reporting bias assessment	14	Describe any methods used to assess risk of bias due to missing results in a synthesis (arising from reporting biases).	6th page 22-23line
Certainty assessment	15	Describe any methods used to assess certainty (or confidence) in the body of evidence for an outcome.	Null

Section and Topic	Item #	Checklist item	Location where item is reported
**RESULTS**			
Study selection	16a	Describe the results of the search and selection process, from the number of records identified in the search to the number of studies included in the review, ideally using a flow diagram.	6th page 25-29line 7th page 1-4 lines and 17th Figure 1
	16b	Cite studies that might appear to meet the inclusion criteria, but which were excluded, and explain why they were excluded.	Null
Study characteristics	17	Cite each included study and present its characteristics.	7th page 6-11lines
Risk of bias in studies	18	Present assessments of risk of bias for each included study.	7th page 16-19lines
Results of individual studies	19	For all outcomes, present, for each study: (a) summary statistics for each group (where appropriate) and (b) an effect estimate and its precision (e.g. confidence/credible interval), ideally using structured tables or plots.	19th Table1
Results of syntheses	20a	For each synthesis, briefly summarise the characteristics and risk of bias among contributing studies.	18th Table1
	20b	Present results of all statistical syntheses conducted. If meta-analysis was done, present for each the summary estimate and its precision (e.g. confidence/credible interval) and measures of statistical heterogeneity. If comparing groups, describe the direction of the effect.	19th Figure 2
	20c	Present results of all investigations of possible causes of heterogeneity among study results.	8th page 8-13lines and 19th Table2
	20d	Present results of all sensitivity analyses conducted to assess the robustness of the synthesized results.	8th page 25-27lines
Reporting biases	21	Present assessments of risk of bias due to missing results (arising from reporting biases) for each synthesis assessed.	8th page 28-30lines
Certainty of evidence	22	Present assessments of certainty (or confidence) in the body of evidence for each outcome assessed.	Null
**DISCUSSION**			

Section and Topic	Item #	Checklist item	Location where item is reported
Discussion	23a	Provide a general interpretation of the results in the context of other evidence.	8th page15-27 lines ,9th page and 10th 1-3lines
	23b	Discuss any limitations of the evidence included in the review.	11thpage8-14lines
	23c	Discuss any limitations of the review processes used.	11th page14-22 lines
	23d	Discuss implications of the results for practice, policy, and future research.	10th page 23-28 lines
**OTHER INFORMATION**			
Registration and protocol	24a	Provide registration information for the review, including register name and registration number, or state that the review was not registered.	4th page 9 line
	24b	Indicate where the review protocol can be accessed, or state that a protocol was not prepared.	Null
	24c	Describe and explain any amendments to information provided at registration or in the protocol.	Null
Support	25	Describe sources of financial or non-financial support for the review, and the role of the funders or sponsors in the review.	11th page 5-7lines
Competing interests	26	Declare any competing interests of review authors.	Null
Availability of data, code and other materials	27	Report which of the following are publicly available and where they can be found: template data collection forms; data extracted from included studies; data used for all analyses; analytic code; any other materials used in the review.	Null

From: Page MJ, McKenzie JE, Bossuyt PM, Boutron I, Hoffmann TC, Mulrow CD, et al. The PRISMA 2020 statement: an updated guideline for reporting systematic reviews. BMJ 2021;372:n71. doi: 10.1136/bmj.n

Table S2. Critical appraisal of the included studies based on the Agency for Healthcare Research and Quality (AHRQ) scale

Items	Min 2018	Zhu 2024	Obayashi 2013	Burns 2022	Obayashi 2022	Helbich 2020	Paksarian 2022
Source of information	**1**	**1**	**1**	**1**	**1**	**1**	**1**
Inclusion /exclusion criteria	**1**	**1**	**1**	**1**	**1**	**1**	**1**
Time period used for identifying patients	**0**	**0**	**0**	**0**	**0**	**1**	**1**
Whether subjects were consecutive if not population-based	**1**	**1**	**1**	**0**	**1**	**1**	**1**
If subjective components were masked to other aspects	**0**	**0**	**0**	**0**	**0**	**0**	**0**
Any assessments for quality assurance	**0**	**0**	**1**	**1**	**0**	**0**	**0**
Any patient exclusions from analysis	**1**	**0**	**0**	**1**	**0**	**1**	**0**
Confounding factors control	**1**	**1**	**1**	**1**	**1**	**1**	**1**
Missing data explanation	**0**	**0**	**1**	**0**	**1**	**1**	**1**
Response rate / completeness of data collection	**1**	**1**	**1**	**1**	**1**	**1**	**1**
Expected follow -up (if any) / percentage of patients for which incomplete data	**1**	**0**	**0**	**1**	**0**	**0**	**0**
Total score	**7**	**5**	**6**	**7**	**6**	**8**	**7**

The methodological quality of the cross-sectional studies was assessed using an 11-item checklist which was recommended by Agency for Healthcare Research and Quality (AHRQ).

An item would be scored '0' if it was answered 'NO' or 'UNCLEAR'; if it was answered 'YES', then the item scored '1'. Article quality was assessed as follows: low quality = 0-3; moderate quality = 4-7; high quality = 8-11.

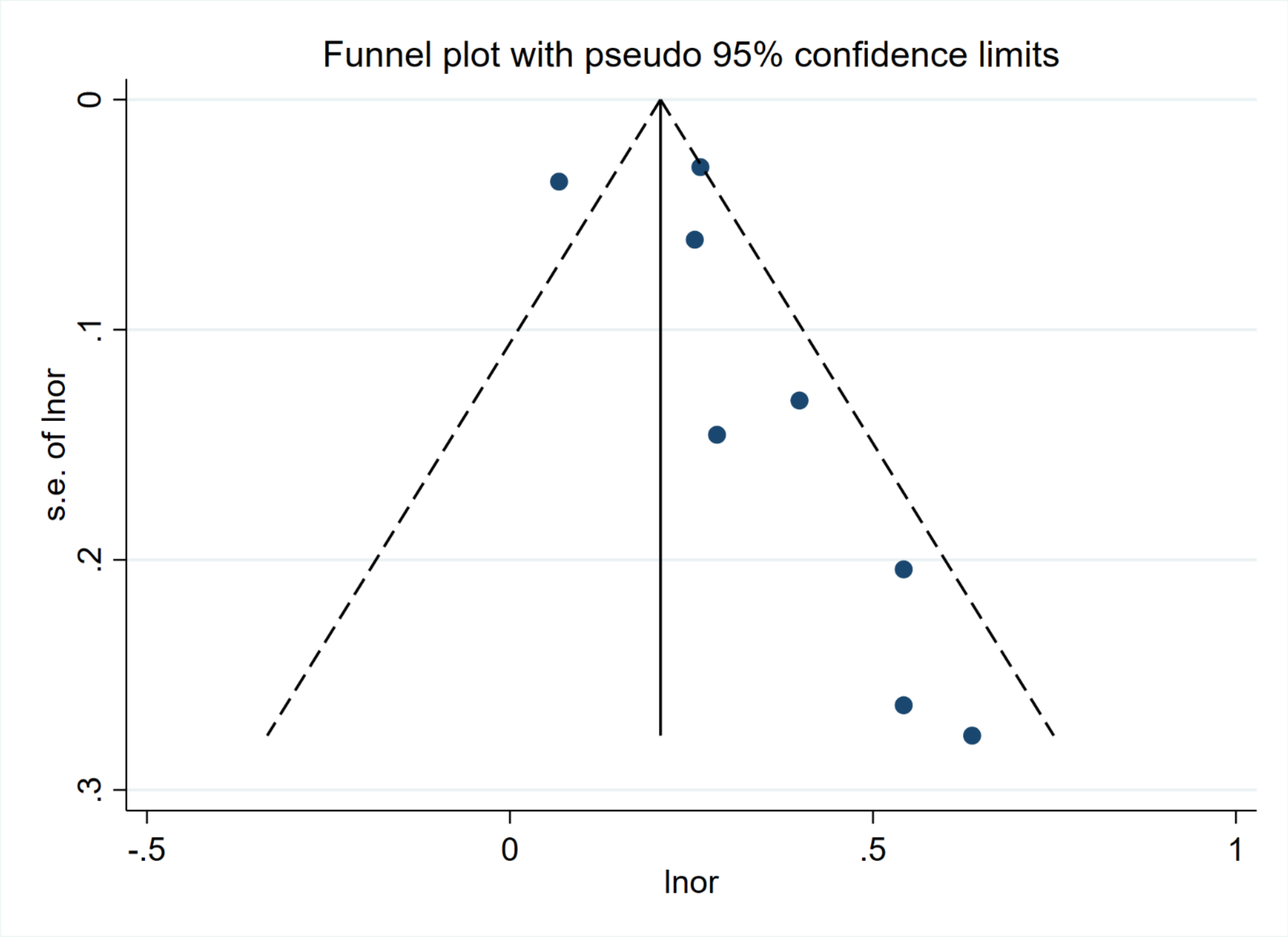

Figure S1. The bias testing results for LAN exposure and depression. LAN – light at night.
